# Human Platelet Lysate Induces Antiviral Responses against Parechovirus A3

**DOI:** 10.3390/v14071499

**Published:** 2022-07-08

**Authors:** Ming-Wei Jan, Chih-Yun Chiu, Jih-Jung Chen, Tsung-Hsien Chang, Kuen-Jer Tsai

**Affiliations:** 1 Institute of Clinical Medicine, College of Medicine, National Cheng Kung University, Tainan 704, Taiwan; davidjan0429@gmail.com; 2 Department of Medical Education and Research, Kaohsiung Veterans General Hospital, Kaohsiung 813, Taiwan; 3 Department and Graduate Institute of Microbiology and Immunology, National Defense Medical Center, Taipei 114, Taiwan; debby861207@gmail.com; 4 Department of Pharmacy, School of Pharmaceutical Sciences, National Yang Ming Chiao Tung University, Taipei 112, Taiwan; jjungchen@nycu.edu.tw; 5 Department of Medical Research, China Medical University Hospital, China Medical University, Taichung 404, Taiwan; 6 Research Center of Clinical Medicine, National Cheng Kung University Hospital, College of Medicine, National Cheng Kung University, Tainan 704, Taiwan

**Keywords:** human platelet lysate, parechovirus-A3, type I interferon, fludarabine

## Abstract

Human platelet lysate (hPL) contains abundant growth factors for inducing human cell proliferation and may be a suitable alternative to fetal bovine serum (FBS) as a culture medium supplement. However, the application of hPL in virological research remains blank. Parechovirus type-A3 (PeV-A3) belongs to *Picornaviridae,* which causes meningoencephalitis in infants and young children. To understand the suitability of hPL-cultured cells for PeV-A3 infection, the infection of PeV-A3 in both FBS- and hPL-cultured glioblastoma (GBM) cells were compared. Results showed reduced PeV-A3 infection in hPL-cultured cells compared with FBS-maintained cells. Mechanistic analysis revealed hPL stimulating type I interferon (IFN) antiviral pathway, through which phospho-signal transducer and activator of transcription 1 (STAT1), STAT2, interferon regulatory factor 3 (IRF3) were activated and antiviral genes, such as *IFN-α*, *IFN-β*, and Myxovirus resistance protein 1 *(MxA*), were also detected. In addition, an enhanced PeV-A3 replication was detected in the hPL-cultured GBM cells treated with STAT-1 inhibitor (fludarabine) and STAT1 shRNA. These results *in vitro* suggested an unexpected effect of hPL-activated type I IFN pathway response to restrict virus replication and that hPL may be a potential antiviral bioreagent.

## 1. Introduction

In the current protocol of in vitro studies, growth medium supplements rely mainly on animal serum because it contains essential growth and attachment factors and abundant nutrients for cell proliferation. Of animal sera, fetal bovine serum (FBS) is widely used as a culture medium supplement in cell-based diagnosis and research [1]. Viruses are intracellular pathogens; FBS-supplemented medium is also commonly used for virus propagation and in studies on virus–host interaction [2,3]. However, apprehensions of product variations, contamination, immunogenicity, and animal welfare limit the application of animal serum in tissue regeneration and biotechnology studies [4,5]. Thus, certain animal serum substitutes, such as human serum (huS) or human platelet lysates (hPL), have been developed [6].

hPL is a new blood-derived biological product made from human platelet concentrate. It is known to contain abundant platelet-derived growth factor (PDGF), epidermal growth factor (EGF), and transforming growth factor-β (TGF-β) [7,8,9]. Other than growth factors, over 1000 proteins, including neurotrophins, angiogenic factors, and antioxidants, were identified by proteomics analysis in platelet lysate [10]. hPL was used as a heterogeneous growth medium supplement for human cell proliferation in cell therapy and has been attracting interest. hPL can also find potentially relevant applications in the field of regenerative medicine. hPL was used in supplements cultured using bone marrow mesenchymal stem cells or adipose tissue mesenchymal stem cells without adverse effects on the immunity system [8,11,12,13]. hPL medium used in mammalian cells or cancer cell growth was also evaluated [14,15]. Thus, hPL was considered a suitable alternative to FBS as a culture medium supplement.

Human parechovirus type 1~type 19 (PeV-A1~A19) belongs to *Picornaviridae* [16]. Most PeV-A infections are asymptomatic or cause mild symptoms, including gastroenteritis or influenza-like illness. PeV-A has been recognized as a significant cause of illness in infants aged < 3 years. PeV-A can cause severe illness, such as meningoencephalitis, seizures or sepsis-like manifestations, or septic shock. In addition to supporting treatment, there is currently no therapy for PeV-A [17,18]. Of the 19 genotypes, PeV-A genotypes 1, 3, and 6 are commonly associated with human disease [19,20]. Accumulated cases suggest that PeV-A3 is a neurotropic virus, which notably causes severe diseases in the central nervous system (CNS) and neonatal sepsis [21,22,23].

It is well known that cells sensing viral infection induce antiviral viral infection factors, such as IFN, IFN-inducible antiviral genes, inflammatory cytokines, and chemokines. In contrast, viruses have evolved several strategies to subvert type I IFN signaling and hinder host antiviral response to maintain viral replication and spread [24]. Our previous research revealed PeV-A1-activated innate immunity in A549 cells, which was not detected in colonic adenocarcinoma-derived T84 cells, indicating cell-type specificity of viral infection-mediated response [25].

The bioactive factors in hPL may regulate cellular signaling responses [10]. The platelet-mediated antiviral effect previously reported found that platelet kinocidin inhibits HIV in T cells [26]. In addition, possible uses of platelets and platelet lysates for various viral infections or COVID-19 pneumonia treatment have recently been suggested [27].

hPL was used as a substitute for FBS in cell culture, and we hypothesized that hPL may be useful for virus propagation in vitro. Thus, this study attempts to examine whether hPL has a similar performance as FBS in the cultivation of fibroblasts. We also determined whether hPL regulates the growth of human glioblastoma cells and the cellular antiviral responses, which would influence the adaption of hPL in PeV-A3 research. The results obtained provide useful information for evaluating whether hPL is a proper substitute for FBS in virus replication.

## 2. Materials and Methods

### 2.1. Cell Line, Serum, and Serum Substitute Preparation

Human glioblastoma cells (DBTRG-05MG, GBM cells) (Bioresource Collection and Research Center, BCRC-60380, Hsinchu, Taiwan) and A549 cells (ATCC: CCL-185) were cultured in RPMI-1640 medium (Gibco, 31800022, Waltham, MA, USA) supplemented with 10% FBS (Gibco, A476680, Waltham, MA, USA), 2.5% hPL or 5% hPL (UltraGRO Helios, research grade, HPCPLCRL50, Atlanta, GA, USA) plus final concentration of 2 U/mL heparin sodium (CCPC, Taipei, Taiwan). Penicillin-streptomycin (P/S, Gibco, 15140122, Waltham, MA, USA) was added to the medium. According to the manufacturers’ datasheet, the hPL was collected from healthy donors at the U.S Food and Drug administration (FDA) and had passed quality control (supplementary information Table S1). Each healthy donor was tested non-reactive for hepatitis B surface antigen (HBsAg), hepatitis C virus (HCV) antibody, human immunodeficiency virus (HIV) antibody, human T-lymphotropic virus antibody (HTLV), Trypanosoma cruzi antibody and negative expression of HIV, HBV, and HCV nucleic acid testing.

### 2.2. Viruses and Reagents

Clinically isolated human parechovirus type 1 (PeV-A1 KVP6, accession no. KC769584) and human parechovirus type 3 (PeV-A3, VGHKS-2007, accession no. KM986843.1) propagation in Vero cells were as previously described [25,28,29]. Dengue virus type 2 (DENV-2) (PL046) isolated from patients with dengue fever was propagated in C6/36 cells [30]. The human Aichi virus (AiV), genome accession no. JX56424 [31], was isolated from a newborn with diarrhea in Taiwan and propagated in Vero cells (ATCC: CCL-81, Manassas, VA, USA). The virus titer was detected with a plaque-forming assay. Both STAT1 inhibitor fludarabine (Selleckchem, S1491, Houston, TX, USA) and interferon-alpha (IFN-α) (Sigma-Aldrich, IF007, St. Louis, MO, USA) were used.

### 2.3. Cell Proliferation Assay

GBM cells (1 × 10^4^) were grown in a 96-well plate with a culture medium supplemented with FBS, 2.5% and 5% hPL. After incubation for 6 h, 24 h, 48 h, and 72 h, cell proliferation was determined using 10 μL CellTiter-Glo luminescent reagent (Promega, G7572, Madison, WI, USA). The luminescent signal was monitored using Synergy HTX (BioTek, Winooski, VT, USA).

### 2.4. RNA Extraction and RT-qPCR

Total RNA from cells was extracted with 1 mL TRIzol reagent (Invitrogen, 15596018, Waltham, MA, USA), followed by the addition of 200 µL 1-Bromo-3-chloropropane (BCP) (Sigma-Aldrich, B9673, St. Louis, MO, USA). The emulsion was centrifuged at 12,000× *g* at 4 °C for 15 min. The RNA in the upper aqueous phase was precipitated with 500 µL isopropanol (Sigma-Aldrich, 278475, St. Louis, MO, USA) and then pelleted by centrifugation at 12,000× *g* at 4 °C for 15 min. The RNA pellet was washed with 1 mL 75% ethanol and then centrifuged at 12,000× *g* for 10 min. The RNA pellet was resuspended in nuclease-free water and measured using a spectrophotometer (Eppendorf, Hamburg, Germany). For RT-qPCR, cDNA was synthesized using of random primer, dNTP with 2 μg total RNA in a total volume of 20 μL by Superscript III Reverse-Transcriptase method (Invitrogen, 18080085, Waltham, MA, USA). The qPCR reaction was conducted with 5 ng cDNA in Fast SYBR Green Master Mix (ThermoFisher, 4385612, Waltham, MA, USA) with 3 μM primers (Genomics, New Taipei City, Taiwan) (supplementary information Table S2). The procedure of qPCR was 95 °C for 3 min for 1 cycle, then 95 °C for 20 s and 60 °C for 30 s for 40 cycles. A melting curve stage was performed from 60 °C to 95 °C in the ABI StepOnePlus Real-Time PCR System (Life Technologies, Waltham, MA, USA).

### 2.5. Immunofluorescence Assay

Mock or PeV-A3, AiV-infected (1 × 10^5^) cells were fixed with 4% paraformaldehyde for 30 min, then permeabilized with 0.5% Triton X-100 in phosphate-buffered saline (PBS) for 10 min, and then blocked with 10% skim milk in PBS for 30 min. The PeV-A3 VP0 and AiV capsid protein VP1 were detected with anti-PeV-A3 VP0 antibody and anti-AiV VP1 antibody (1:500), respectively [2,3,32,33], followed by Alexa 568-conjugated anti-rabbit IgG antibody (1:1000; ThermoFisher Scientific), each incubated for 1 h at 25 °C. Cell nuclei were stained with 300 nM 4′,6′-diamidino-2-phenylindole (DAPI) for 10 min. Fluorescence signals were observed and captured under a fluorescence microscope.

### 2.6. Immunoblotting Analysis

The cells were lysed in protein lysis buffer (2% SDS, 50 mM Tris-HCl pH = 7.5) containing protease and phosphatase inhibitor (ThermoFisher, 78442, Waltham, MA, USA). The cell lysates were homogeneously sonicated. The protein concentration was calculated using DC Protein Assay Reagent (Bio-Rad, 500116, Hercules, CA, USA). Then, 60 µg protein lysates were separated in 10% SDS-PAGE and transferred to the PVDF membrane (Millipore, IPVH00010, Burlington, MA, USA). PVDF membrane was covered with blocking buffer (10% skim milk) for 1 h and incubated with the primary antibody in 1:1000 dilution in 5% skim milk/TBST at 4oC overnight. The antibodies were anti-PeV VP0 (LTK BioLaboratories, Taoyuan, Taiwan) [2,3], anti-phospho STAT2 (Cell Signaling, 88410, Danvers, MA, USA), anti-total STAT2 (Cell Signaling, #72604), anti-phospho STAT1 (Cell Signaling, #9167), anti-total STAT1 (Cell Signaling, #14994), anti-phospho IRF3 (Abcam, #ab76493, Cambridge, England), anti-total IRF3 (Cell Signaling, #11904), anti-phospho NFκB p65 (Abcam, #ab86299), anti-total NFκB p65 (Cell Signaling, #8242) and anti-GAPDH (Proteintech, 60004-1-Ig, Rosemont, IL, USA). Secondary antibodies were HRP-conjugated rabbit anti-mouse IgG (LEADGENE, 20112, Tainan, Taiwan) and HRP-conjugated goat anti-rabbit IgG (LEADGENE, 20202, Tainan, Taiwan). The images were acquired from the BioSpectrum imaging system (UVP Analytik Jena, Jena, Germany) using the WesternBright ECL kit (Advansta, K-12045-D20, Menlo Park, CA, USA).

### 2.7. STAT1 Knockdown

GBM cells were transfected with STAT1 knockdown shRNA and negative control shRNA for 48 h; then, the cells were infected with PeV-A3 for 48 h. STAT-1 knockdown efficiency was analyzed by immunoblotting with an anti-STAT1 antibody. The PeV-A3 was detected by RT-qPCR and immunoblotting.

### 2.8. Statistical Analysis

Statistical analysis was performed using Prism 9 (Graphpad, San Diego CA, USA). Student’s *t*-test was used for quantitative parameters. The results were expressed as mean ± standard deviation (SD). *p*-value of < 0.05 was considered statistically significant.

## 3. Results

### 3.1. Comparison of GBM Growth Status between hPL and FBS Culture Mediums

**Figure 1:**
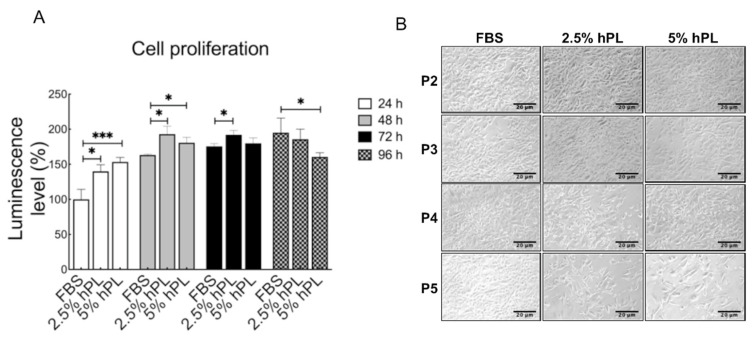
Cell proliferation analysis and morphology observation in GBM cells cultured with FBS and hPL mediums. (**A**) Cell-Titer Glo assay was investigated in GBM cells maintained in FBS, 2.5% and 5% hPL mediums for 24 h–96 h. Data are mean ± SD of five independent experiments. Student’s *t*-test, *, *p* < 0.05, ***, *p* < 0.001. (**B**) The morphology of GBM cells cultivated from passage 2 to passage 5 with FBS, 2.5% and 5% hPL culture mediums. GBM cells were subcultured every 4 days under serum or serum substitute culture mediums, and subcultivation ratio of 1:4 was determined. Scale bar = 20 μm.

To evaluate the feasibility of hPL as an FBS substitute in the GBM cell culture medium, the GBM proliferation activity of hPL and FBS were compared. According to the manufacturer’s instruction, 2.5–5% hPL has a similar performance as 10% FBS; therefore, GBM cells were grown in 10% FBS, 2.5%, and 5% hPL growth mediums, and the cell proliferation at 24 h, 48 h, 72 h, and 96 h were determined. Compared with FBS, 2.5% and 5% hPL showed slightly higher levels of cell proliferation activity in GBM cells at 24 h and 48 h. The 5% hPL-promoted cell growth was retarded at 96 h (Figure 1A). The effect of hPL on cell proliferation was also evaluated and compared with FBS in other cell types; the 2.5% and 5% hPL enhanced the growth activity of HeLa cells at 24 h and then decelerated during the incubation period; in addition, Vero cells proliferation was promoted by hPL throughout the incubation (Supplementary Information Figure S1). The results suggested a cell-type-dependent effect of hPL-regulated cell proliferation. Next, GBM cells were subcultured every four days, and the growth status in the series of passaging cells with hPL and FBS culture mediums was observed. The early passages showed no morphological difference in cells supplemented with FBS, 2.5% and 5% hPL mediums. However, in the fifth passage of GBM cells, 2.5% and 5% hPL-cultured cells demonstrated poor proliferative response compared with FBS-cultured cells (Figure 1B). These findings revealed lower proliferation activity in GBM cells undergoing long-term culture and multiple passages with hPL medium than with FBS medium. Thus, this study used short-term cultivation and early passages in the viral infection analysis.

### 3.2. Downregulation of Viral Replication in hPL Culture Medium-Maintained GBM Cells

**Figure 2:**
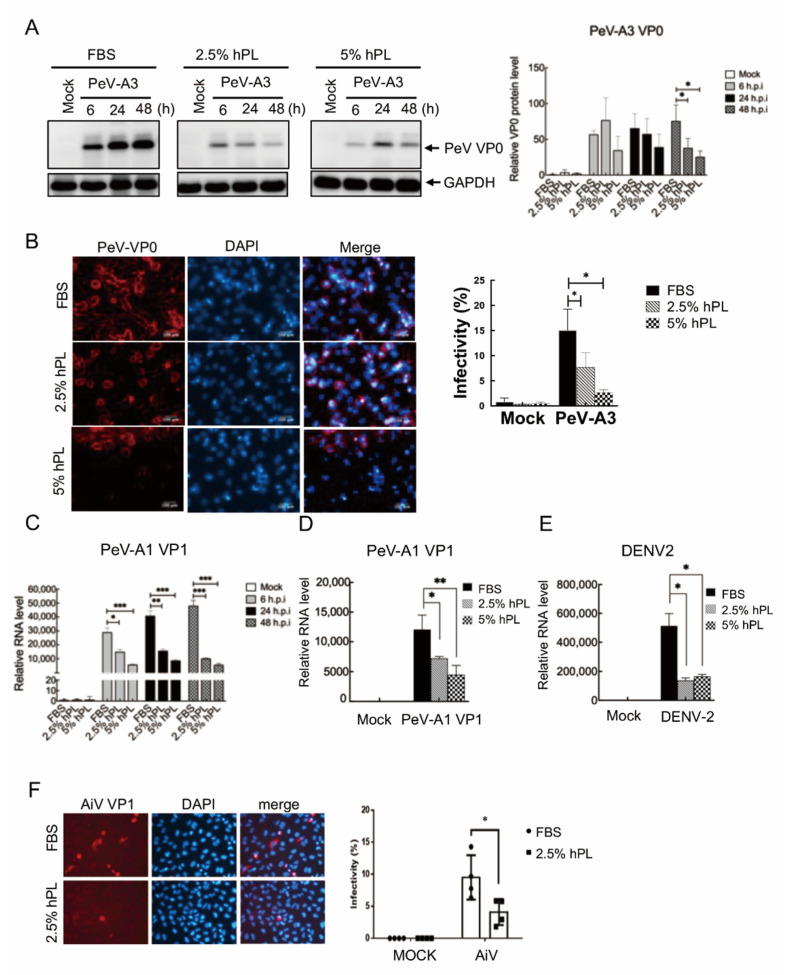
GBM cells infected with PeV-A3 and other viruses showing reduced viral protein and viral RNA expression under hPL medium compared with FBS medium. (**A**) GBM cells were infected with PeV-A3 at MOI = 1 for 6 h, 24 h, and 48 h under FBS- and hPL-supplemented mediums. Protein lysates were examined by immunoblotting using anti-PeV-VP0 and anti-GAPDH (left panels). Quantification of protein expression was normalized to GAPDH. Data are mean ± SD of three independent experiments. Student’s *t*-test, *, *p* < 0.05 (right panel). (**B**) BGM cells were infected with PeV-A3 at MOI = 1 for 48 h. Cells were then stained with anti-PeV-VP0 antibody followed by goat anti-rabbit IgG secondary antibody. Scale bar = 100 μm (left panels). Quantified PeV-A3 infected GBM cells of immunofluorescence assay are shown. Data are mean ± SD. Student’s *t*-test, *, *p* < 0.05 (right panel). (**C**,**D**) RNA level of PeV-A3 VP1 and PeV-A1 VP1 genes under serum and serum substitute mediums. mRNA levels of PeV-A3 VP1 and PeV-A1 VP1 were measured and normalized using GAPDH. Student’s *t*-test, *, *p* < 0.05; **, *p* < 0.01, ***; *p* < 0.001 compared with FBS. (**E**) GBM cells were infected with DENV-2 at MOI = 1 for 24 h under FBS- and hPL-cultured mediums. mRNA level of DENV-2 was measured and normalized using GAPDH. Data are mean ± SD of three independent experiments. Student’s *t*-test, *, *p* < 0.05. (**F**) A549 cells were infected with AiV at MOI = 1 for 48 h. Cells were then stained with anti-AiV VP1 antibody followed by goat anti-rabbit IgG secondary antibody. Scale bar = 100 μm. (left panels). Quantified AiV VP1 infection levels of immunofluorescence assay of A549 cells are shown as scattered plots. Data are mean ± SD. Student’s *t*-test, *, *p* < 0.05 (right panel).

With the hPL-supported GBM cell growth determined, the efficiency of virus replication in both hPL- and FBS-cultured GBM cells were evaluated. GBM cells in FBS, 2.5% and 5% hPL mediums were infected with PeV-A3 at MOI = 1 for 6 h, 24 h, and 48 h (h.p.i.). Immunoblotting assay results showed downregulation in the level of PeV-VP0 proteins in both 2.5% and 5% hPL mediums when compared with the FBS-cultured group (Figure 2A, left panels). Statistical analysis results indicated that hPL significantly decreased the protein level of PeV-VP0 in GBM cells at 48 h post-infection (h.p.i.) (Figure 2A, right panels). We further conducted the immunofluorescence assay of PeV-A3 infection in GBM cells. Compared with FBS, hPL-cultured GBM cells showed lower infectivity of PeV-A3 (Figure 2B). PeV-A3 gene replication measured with RT-qPCR also showed significant inhibition in PeV-A3 VP1 gene expression level in 2.5% or 5% hPL-cultured GBM cells compared with FBS-supported GBM cells (Figure 2C). These results suggested that PeV-A3 replication was less efficient in hPL-cultured cells than in FBS-supported cells.

To confirm the hPL-mediated inhibition of virus replication, additional virus infections were conducted. GBM cells were infected with PeV-A1 or dengue virus type 2 (DENV-2) at MOI = 1, and the viral gene replication was measured. Results showed significant inhibition of viral replication in GBM cells with 2.5% and 5% hPL-supplemented growth mediums (Figure 2D,E). Moreover, another picornavirus, AiV [34], which causes gastroenteritis, was used in the analysis. The immunofluorescence assay of AiV infection was conducted. Compared with FBS medium-maintained A549 cells, hPL-cultured A549 cells had lower infectivity of AiV (Figure 2F). These results indicated an unrevealed antiviral activity of hPL.

### 3.3. hPL Induced Type I Interferon Response in GBM Cells

**Figure 3:**
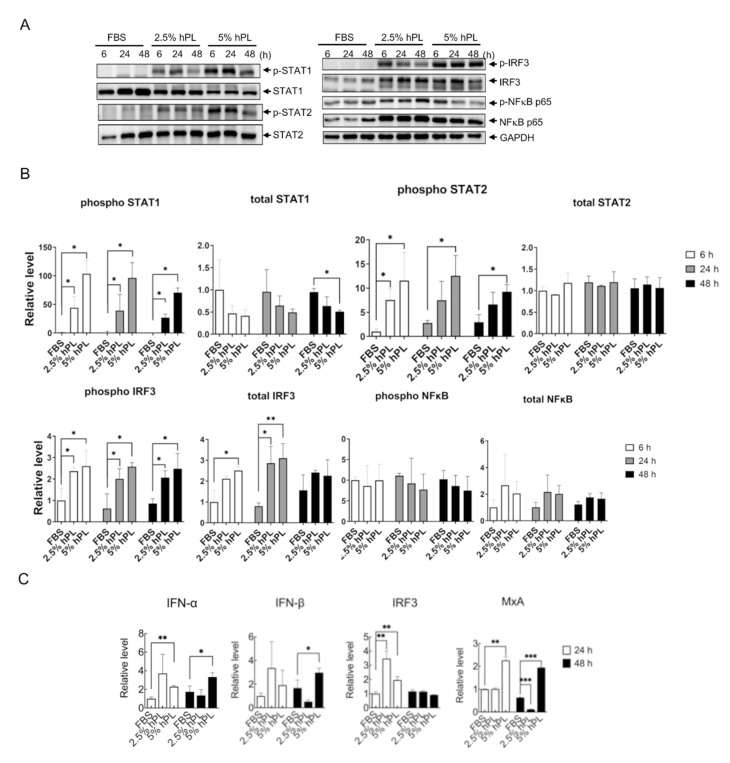
hPL-induced innate immunity response type I interferon and antiviral-related genes on GBM cells. (**A**) GBM cells were cultured under FBS, 2.5% and 5% hPL for 6 h, 24 h, and 48 h. The cell protein lysates were employed to analyze the innate immunity pathways by immunoblotting using anti-phospho-STAT1, anti-total STAT2, anti-phospho-STAT2, anti-total STAT2, anti-phospho-IRF3, anti-total IRF3, anti-phospho-NFκB, anti-total NFκB p65, and anti-GAPDH. (**B**) Quantification of protein expressions was normalized to GAPDH. Data are mean ± SD of three independent experiments. Student’s *t*-test, *, *p* < 0.05; **, *p* <0.01 compared with GBM cells supplemented with FBS. (**C**) GBM cells were cultured under FBS, 2.5% and 5% hPL mediums for 24 h and 48 h. The mRNA level of antiviral-related genes *IFN-α*, *IFN-β*, *IRF3*, and *MxA* were confirmed by qPCR. The level of transcripts was normalized to GAPDH. Data are mean ± SD of three independent experimental samples. Student’s *t*-test, *, *p* < 0.05; **, *p* < 0.01; ***, *p* < 0.001 compared with FBS.

hPL contains abundant growth factors and cytokines [7,8,9]. The hPL-mediated virus reduction may be attributed to the activation of cellular antiviral responses in GBM cells cultured with hPL-supplemented growth medium. In particular, it would be important to elucidate whether IFN antiviral response was correlated with the hPL-mediated restriction of viral replication [35]. Type I IFN-signaling pathway was analyzed by determining the activation of STAT1/2, IRF3 and NFκB p65 in GBM cells cultured with FBS- or hPL-supplemented medium for 6 h, 24 h, and 48 h. Immunoblotting assay results showed activation of type I IFN-signaling proteins of phospho-STAT1, phospho-STAT2, and phospho-IRF3 in GBM cells cultivated in 2.5% and 5% hPL- supplemented medium compared with FBS-supplemented medium (Figure 3A, left panels); the total protein amount of IRF3 and NFκB p65 were increased in hPL-cultured GBM cells (Figure 3A, right panels). The protein level was also quantified and statistically analyzed. Compared with FBS, hPL significantly increased the level of phospho-STAT1, phospho-STAT2, phospho-IRF3, and total IRF3 (Figure 3B). Moreover, significant induction of gene expression of *IFN-α, IFN-β, IRF3,* and *MxA* was detected in GBM cells with a 5% hPL-supplemented medium (Figure 3C). These data suggested that activation of cellular antiviral response in GBM cells incubated with hPL might contribute to virus restriction.

### 3.4. hPL Enhanced IFN Response in PeV-A3-Infected GBM Cells

**Figure 4:**
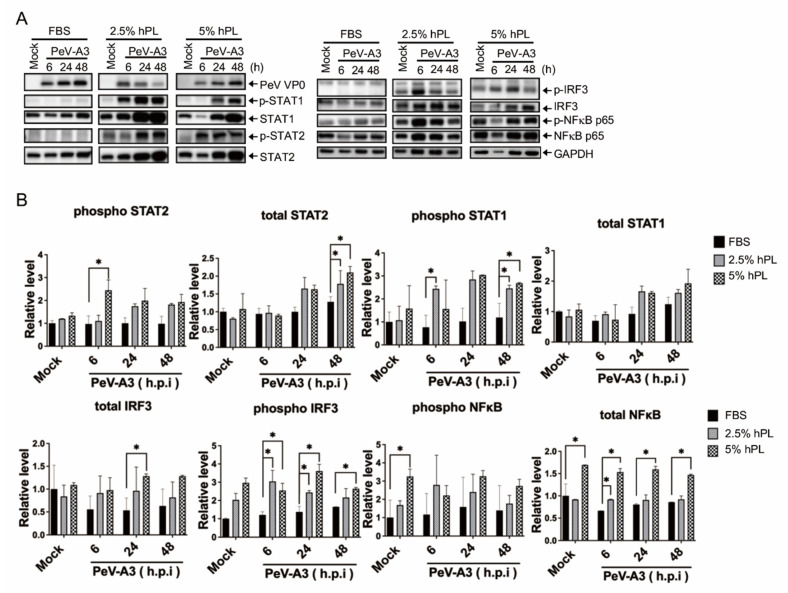
hPL-supplemented growth medium-induced expression of IFN-signaling pathway under PeV-A3 infection of GBM cells. (**A**) GBM cells were infected with PeV-A3 at MOI = 1 for 6 h, 24 h, and 48 h under FBS- or hPL-supplemented growth mediums. Cellular protein lysates were extracted to analyze IFN-signaling pathway by immunoblotting using anti-PeV VP0, anti-phospho-STAT1, anti-total STAT1, anti-phospho-STAT2, anti-total STAT2, anti-phospho-IRF3, anti-total IRF3, anti-phospho-NFκB, anti-total NFκB p65, and anti-GAPDH. (**B**) Quantification of different protein expressions was normalized to GAPDH. Data are mean ± SD of three independent samples. Student’s *t*-test, *, *p* < 0.05 compared with FBS.

To further understand the antiviral response in hPL-cultured GBM cells after PeV-A3 infection, the FBS and hPL medium-cultured GBM cells were infected with PeV-A3 for 6 h, 24 h, and 48 h. Immunoblotting assay results showed that PeV-A3 failed to induce STAT1, STAT2, IRF3, and NFκB p65 activation in FBS medium-maintained GBM cells; in contrast, phosphorylation of STAT1, STAT2, IRF3, and total NFκB p65 were detected in hPL-medium-maintained GBM cells with PeV-A3 infection (Figure 4A). Significantly enhanced levels of signaling proteins in hPL-medium-cultured GBM cells were also observed (Figure 4B).

**Figure 5:**
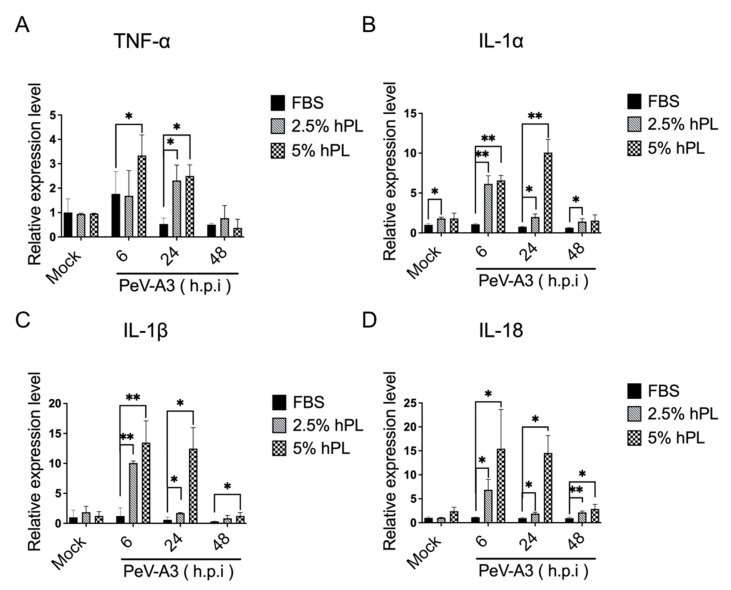
(**A**–**D**) hPL-supplemented growth medium-induced inflammatory cytokines under PeV-A3 infection of GBM cells. Inflammatory cytokines IL-1α, IL-1β, IL18, and TNF-α were analyzed using real-time qPCR. Data are mean ± SD of three independent samples. Student’s *t*-test, *, *p* < 0.05; **, *p* < 0.01 compared with FBS.

This study then assessed the expression of pro-inflammatory cytokines in PeV-A3-infected GBM cells cultured with an FBS- or hPL-supplemented medium. High levels of TNF-α, IL-1α, IL-1β, and IL-18 gene expressions were detected in the 2.5% and 5% hPL medium-cultured GBM cells with PeV-A3 infection. These results suggested that for cells cultured in an hPL growth medium, the increased level of these inflammatory genes may decrease viral replication (Figure 5A–D).

### 3.5. STAT1 Inhibitor Increased PeV-A3 Infection in hPL Medium-Cultured GBM Cells

**Figure 6:**
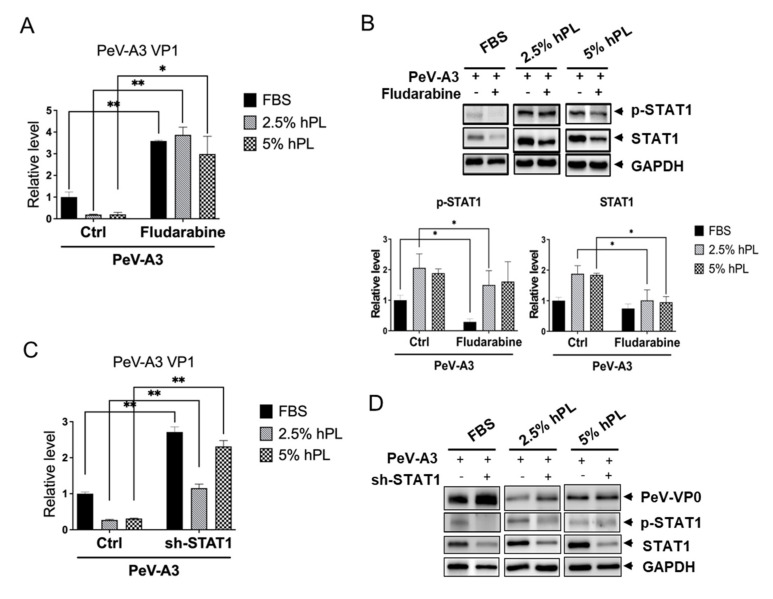
Enhanced PeV-A3 VP1 gene expression under blockade of STAT1 on PeV-A3 infection of GBM cells. (**A**) GBM cells were pre-treated with fludarabine 50 µM for 30 min under FBS or hPL medium. After treatment, GBM cells were then infected with PeV-A3 at MOI = 1 for 6 h. Expression of PeV-A3 VP1 genes was measured using real-time qPCR. The level of PeV-A3 VP1 was normalized to GAPDH. Data are mean ± SD of three independent samples. Student’s *t*-test, *, *p* < 0.05; **, *p* < 0.01 compared with PeV-A3-infected GBM cells under FBS or hPL mediums. (**B**) GBM cells were pre-treated with fludarabine 50 µM for 30 min under FBS or hPL mediums. After treatment, GBM cells were infected with PeV-A3 at MOI = 1 for 6 h. Cellular protein lysates were subjected to immunoblotting with anti-phospho-STAT1, anti-total-STAT1, and anti-GAPDH (upper panels). Quantification of protein expression levels of PeV-VP0, total-STAT1, and phospho-STAT1 was normalized to GAPDH. Data are mean ± SD of three independent experiments. Student’s *t*-test, *, *p* < 0.05 (lower panels). (**C**) The STAT-1 was knockdown by shRNA transfection; the cells were then infected by PeV-A3 at MOI = 1 for 48 h. The expression level of PeV-A3 VP1 RNA was measured by RT-qPCR. Data are mean ± SD of three independent samples. Student’s *t*-test, **, *p* < 0.01 compared with PeV-A3-infected GBM cells under FBS or hPL mediums. (**D**) The protein expression of PeV-A3 VP0, STAT1, phospho-STAT1, and GAPDH were detected by immunoblotting assay.

Results shown above revealed that activated type I IFN response in GBM cells with hPL growth medium might interfere with the viral infection. We presumed that blocking the hPL-induced STAT1 enhanced PeV-A3 replication. Fludarabine is a STAT1 inhibitor, and it suppresses phosphorylation of STAT1 [36]. Fludarabine is used as a common chemotherapy drug for the treatment of chronic lymphocytic leukemia [37]. Thus, whether blocking STAT1 activity with fludarabine [33] could increase PeV-A3 infection was investigated. The cytotoxicity of fludarabine was tested in FBS- and hPL-cultured cells, and the dose of 50 µM without cytotoxicity was used (Supplementary Information Figure S2). FBS- and hPL-cultured GBM cells were pre-treated with or without fludarabine and then infected with PeV-A3 at MOI = 1 for 6 h. Treatment of fludarabine in PeV-A3-infected GBM cells showed a relatively higher level of PeV-A3 VP1 gene expression than those not treated with fludarabine. Enhanced viral gene replication by fludarabine was observed in cells with FBS or 2.5% and 5% hPL mediums. Over three-fold increase in PeV3 VP1 gene was detected in 2.5% hPL-cultured cells than in FBS-cultured cells (Figure 6A). Similarly, inhibition of hPL-mediated SATA1 activation enhanced DENV2 5′UTR gene replication was detected (Supplementary Information Figure S3). The STAT1 inhibition effect of fludarabine was evaluated using an immunoblotting assay, and quantitation data showed a reduction of phospho-STAT1 and total-STAT1 expression in cells treated with fludarabine (Figure 6B). These results suggested that fludarabine reduced the hPL-induced type I IFN-related protein STAT-1 expression and upregulated the PeV-A3 viral RNA expression. To further confirm the hPL antiviral effect through SATA1 activation, the PeV-A3 replication was detected in STAT-1 knockdown GBMs. We detected the increased level of PeV-A3 VP1 RNA in STAT1 knockdown cells (Figure 6C), which suggested that STAT1 activity played a critical role in hPL-mediated virus inhibition.

## 4. Discussion

hPL is considered a suitable substitute for FBS for different types of cell cultivation [38,39,40]. This study examined whether hPL is applicable in the cell-based model of viral infection. The infection results showed that hPL-cultured GBM cells were not efficiently infected by PeV-A3, PeV-A1, DENV-2, and AiV compared with FBS-cultured cells. Mechanistic analysis revealed that hPL stimulated type I IFN antiviral machinery, activating STAT1/2, IRF3, and NF-κB p65. A high level of antiviral gene expression, such as *IFN-α*, *IFN-β*, and *MxA*, was detected in hPL-cultured cells but not in FBS-cultured cells. Moreover, PeV-A3 infection induced a higher level of type I IFN pathway activation and inflammatory cytokine expression (TNF-α, IL-1α, IL-1β, and IL-18) in hPL-cultured GBM cells than in FBS-maintained cells. These data suggested that hPL stimulated a potential antiviral response against virus infection *in vitro*. This hypothesis was further demonstrated by fludarabine treatment, which blocked STAT1 activity from increasing virus replication in hPL-cultured GBM cells. The present results indicated that hPL may be a potential antiviral bioreagent; however, using commercial hPL as an FBS substitute for cell culture-based viral infection should be further assessed.

Compared with FBS, hPL was found to contain a higher concentration of growth factors that benefit cell growth of the common laboratory mammalian cells, such as Vero cells and Hep-2 cells [15]. The present growth analysis results of GBM cells echo this finding (Figure 1A, Supplementary Information Figure S1). However, compared with FBS-cultured cells, hPL-cultured GBM cells, after passaging for five generations, showed reduced growth activity and morphology of sharper elongated spindle (Figure 1B). To our knowledge, this is the first finding on the downregulated proliferation of cells after long-term exposure to or multiple passaging with hPL. Although the underlying mechanism requires further investigation, long-term exposure to hPL-mediated cytokine release or stimulation may cause cellular senescence [41,42]. hPL contained high concentrations of PDGF, TGF-β, EGF, and bone morphogenetic proteins (BMP) [8,43]. TGF-β and TGF family gene BMP4 could attenuate brain tumor stem cell proliferation through the SMAD pathway [44,45]. Another understanding of intrinsic IFN activity in GBM cells cultivated with hPL may interfere with the continuous growth in comparison with cultivation with FBS. Thus, the role of TGF-β and BMP4 in GBM cells under long-term hPL medium exposure merits further investigation.

This study used the early passage of hPL-cultured GBM cells for viral infection. The findings revealed an unexpected antiviral effect of hPL. Lower level of PeV-A3, PeV-A1, and DENV-2 infection was detected in hPL-cultured GBM cells and less infectivity of AiV in hPL-cultured A549 cells (Figure 2). Further mechanistic analysis indicated that hPL-induced cellular innate immune response played a critical role against virus infection (Figure 3 and Figure 6). The previous study found that platelet factor 4 (PF-4/CXCL4) was a potential antiviral platelet kinocidin to inhibit HIV in T cells [26]. Possible uses of platelets and platelet lysates for various viral infections or COVID-19 pneumonia treatment have recently been suggested since it acts as a regenerative immunomodulator against COVID-19 by introducing growth factors, cytokines, and proteins at supraphysiological levels to attenuate changes caused by immune responses in allogeneic effects [27]. These reports partly support our finding of hPL against PeV-A3 infection. An appropriate concentration of heparin had to be added to hPL growth mediums for cell culture [46]. Heparin inhibition of flaviviruses infection was reported [47]; hence, the possible antiviral effect of heparin in an hLP-containing medium cannot be ruled out. Thus, optimized use of heparin in hPL-supported cell culture has been suggested [15]. Moreover, only one resource of hPL was used in this study, and different resources or prepared hPL should be further evaluated.

This study found that PeV-A3 failed to activate STAT-1, STAT-2, and IRF3 signaling in cells cultured in the FBS growth medium; in contrast, these transcription factors were activated in PeV-A3-infected GBM cells with an hPL-containing medium (Figure 4). Higher levels of PeV-A3-induced inflammatory cytokines were detected in hPL-cultured cells but not in FBS-cultured cells (Figure 5). These results echoed in part that PeV-A3 triggered inflammatory responses in airway epithelial cells [48]. Platelet lysate contains abundant growth factors, chemokines, and bioactive mediators, and it is reasonable to speculate that these mediators may contribute to the inflammatory cytokine expression in hPL-cultured GBM. However, determination of the cytokine expression of IL-1β, IL-2, IL-6, IL-10, IL-12p70, 1L-17A, TNF-α, and IFN-γ in prepared platelet lysate revealed that cytokines were present only in extremely low concentrations [49]. We suggest that this low level of inflammation cytokines in hPL may contribute to viral infection-triggered cellular responses.

Whether using different culture systems to investigate virus and host interaction may yield completely different results merits exploration. Therefore, a proper animal infection model should be considered for investigating the regulatory effect of hPL in viral infection and antiviral innate immunity.

We demonstrated that fludarabine effectively increased viral replication during PeV-A3 infection. STAT1 inhibitor, Fludarabine, -treated FBS- and hPL-cultured GBM cells showed reduced levels of phospho-STAT1 and elevated levels of STAT1 protein under PeV-A3 infection; similar results were detected in STAT1 knockdown cells (Figure 6). Taken together, these results confirmed that hPL inhibits viral infection by activation of the cellular antiviral STAT1 pathway. Another study showed that fludarabine inhibits enterovirus type 71 (EV-71), SFTS Phlebovirus, and ZIKA virus infection in human astrocytes and human microglia cells [50]; however, the antiviral mechanism was not investigated. Whether fludarabine protects or destroys infected neuronal cells should be further investigated and verified in animal models.

In conclusion, the present study suggested that the commercial hPL activated the type I IFN response against viral infection *in vitro*. Thus, hPL may be a potential antiviral bioreagent that merits further evaluation. The antiviral response when using commercial hPL as an FBS substitute for cell culture-based viral infection merits in-depth evaluation.

## Data Availability

The data presented in this study are contained within the article.

## Supplementary Materials

The following supporting information can be downloaded at: https://www.mdpi.com/article/10.3390/v14071499/s1. Figure S1: Cell proliferation assay in HeLa and Vero cells cultured with FBS and hPL mediums. Figure S2: Cytotoxicity assay of fludarabine. Figure S3: Enhanced DENV-2 5’UTR gene expression under blockade of STAT1 on DENV-2 infection of GBM cells. Table S1: Quality control of human platelet lysate. Table S2. q-PCR primers used in this study.
