# Supplementary material for: Human Platelet Lysate Induces Antiviral Responses against Parechovirus A3
Source: Viruses. 2022 Jul 8;14(7):1499. doi: 10.3390/v14071499 (PMC9316291; doi:10.3390/v14071499)
Supplement: Supplementary file 1 [file viruses-14-01499-s001.zip › viruses-1646252-supplementary.pdf]

Supplementary information

Table S1. Quality control of human platelet lysate.

Item	Specification	Result
Appearance	Yellow to Buff liquid	Yellow to Buff liquid
Total protein	4.0-8.0 g/dL	6.2 g/dL
Endotoxin	≤ 10 EU/ml	< 0.01 EU/ml
Sterility	Pass	Pass
Mycoplasma	Negative	Negative
pH	Tested	7.7
Osmolality	275-355 mOsM/kg	315 mOsM/kg

Table S2. q-PCR primers used in this study.

Gene	Sequences (5’ - 3’)
DENV-2 5’UTR	F: AGT TGT TAG TCT ACG TGG ACC GA<br>R: CAA GAG AGG GAC CCA CAT CG
Gapdh	F: TGC ACC ACC AAC TGC TTA GC<br>R: GGC ATG GAC TGT GGT CAT
Ifn-a	F: CCT CGC CCT TTG CTT TACT G<br>R: CAG AGA GCA GCT TGA CTT GCA
Ifn-b	F: TGA GCA GTC TGC ACC TGA AA<br>R: GCT TGA AGC AAT TGT CCC GT
Il-1a	F: TGT ATG TGA CTG CCC AAG ATG AA<br>R: TGG GTA TCT CAG GCA TCT CCT T
Il-1b	F: ATG GCT TAT TAC AGT GGC AA<br>R: GTC GGA GAT TCG TAG CTG GA
Il-18	F: TCT TCA TTG ACC AAG GAA ATC GG<br>R: TCC GGG GTG CAT TAT CTC TAC
Irf3	F: TTC CCG GGA GGG ATA AGC<br>R: GGG CAG AGC GGA AAT TCC
Irf7	F: TCC CCA CGC TAT ACC ATC TAC CT<br>R: ACA GCC AGG GTT CCA GCT T
Mxa	F: GCT ACA CAC CGT GAC GGA TAT GG<br>R: CGA GCT GGA TTG GAA AGC CC

Figure S1

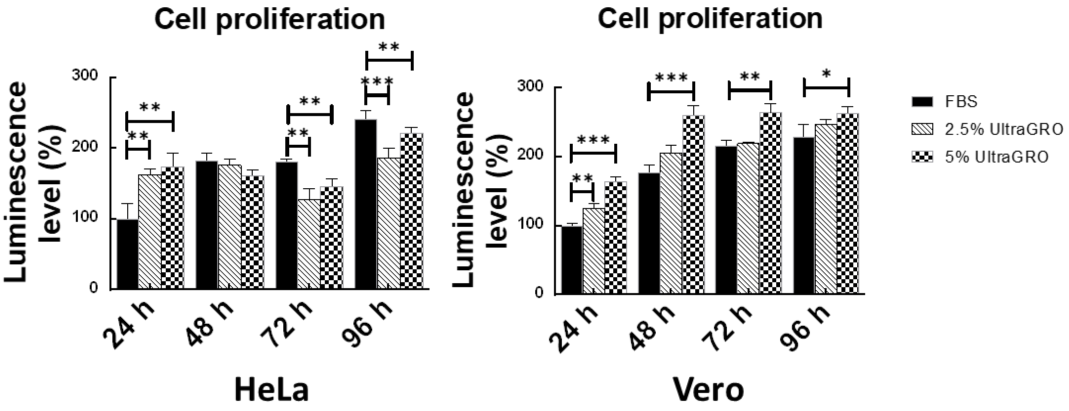

Figure S1. Cell proliferation assay in HeLa and Vero cells cultured with FBS and hPL mediums. (A) Cell-Titer Glo assay was investigated in HeLa (left panel) and Vero (right panel) cells maintained in FBS, 2.5% and 5% hPL mediums for 24 h - 96 h. Data are mean ± SD of five independent experiments. Student's t-test, *, $p < 0.05$, **, $p < 0.01$, ***, $p < 0.001$.

Figure S2

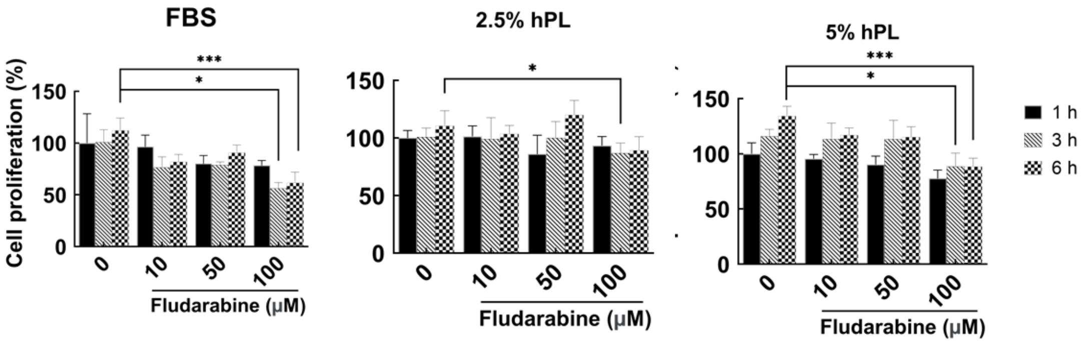

Figure S2. Cytotoxicity assay of fludarabine. FBS or hPL growth medium-cultured GBM cells were treated with 10, 50, 100 µM fludarabine for 1, 3, 6 h. Cell-Titer Glo assay determined the GBM cellular proliferation. Data are mean ± SD of three independent samples. Student's t-test, *, $p < 0.05$; ***; $p < 0.001$ compared.

Figure S3

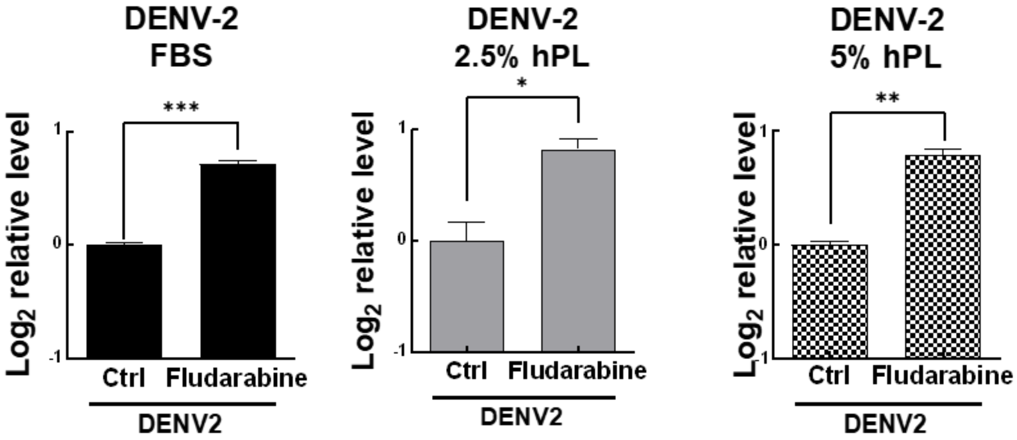

Figure S3. Enhanced DENV-2 5'UTR gene expression under blockade of STAT1 on DENV-2 infection of GBM cells. (A) GBM cells were pre-treated with fludarabine 50 μM for 30 min under FBS or hPL medium. After treatment, GBM cells were then infected with DENV-2 at MOI = 2.5 for 6 h. Expression of DENV-2 5'-UTR gene were measured using real time qPCR. The level of DENV-2 5'-UTR was normalized to GAPDH. Data are mean ± SD of three independent samples. Student's t-test, *, $p < 0.05$; **, $p < 0.01$ compared with PeV-A3-infected GBM cells under FBS or hPL mediums.
